# Microbiome profiling of uncinate tissue and nasal polyps in patients with chronic rhinosinusitis using swab and tissue biopsy

**DOI:** 10.1371/journal.pone.0249688

**Published:** 2021-04-08

**Authors:** Sung-Woo Cho, Dong-Young Kim, Sungmi Choi, Sungho Won, Hye-Ryun Kang, Hana Yi

**Affiliations:** 1 Department of Otorhinolaryngology, Seoul National University Bundang Hospital, Seoul National University College of Medicine, Seoul, Republic of Korea; 2 Department of Otorhinolaryngology, Seoul National University Hospital, Seoul National University College of Medicine, Seoul, Republic of Korea; 3 Institute for Biomaterials, Korea University, Seoul, Republic of Korea; 4 Department of Public Health Science, Seoul National University, Seoul, South Korea; 5 Department of Internal Medicine, Seoul National University Hospital, Seoul National University College of Medicine, Seoul, Republic of Korea; 6 Institute of Allergy and Clinical Immunology, Seoul National University Medical Research Center, Seoul National University College of Medicine, Seoul, Korea; 7 Interdisciplinary Program in Precision Public Health, Korea University, Seoul, Republic of Korea; 8 School of Biosystem and Biomedical Science, Korea University, Seoul, Republic of Korea; Kyungpook National University, REPUBLIC OF KOREA

## Abstract

Chronic rhinosinusitis (CRS) is characterized according to the presence or absence of nasal polyps (NPs) and displays nasal microbiota dysbiosis. However, optimal sampling methods of the nasal microbiome in CRS have not been identified. We aimed to assess the microbial composition in patients with CRS, comparing different sampling methods (swab and tissue biopsy), tissue types (uncinate tissue and NP), and disease subtypes. Samples were obtained by swabbing the middle meatus and taking a biopsy of uncinate tissue (UT) in patients with CRS with (CRSwNP, N = 8) or without NP (CRSsNP, N = 6) and controls (N = 8). NPs were also harvested in CRSwNP. DNAs were extracted from fifty-two samples and analyzed by 16S rRNA gene amplicon sequencing. As a result, a great interpersonal variance was observed in nasal swabs, while UT samples presented distinct microbiome with low inter-personal differences. Moreover, the UT microbiomes were further differentiated into three clusters which are associated with disease status (control, CRSsNP, and CRSwNP). Compared to UT, NP revealed a unique microbiome profile with significantly less bacterial diversity. *Prevotella* was the genus whose abundance was negatively correlated with disease severity in NP. In conclusion, tissue samples are better specimens than nasal swabs for assessing the microbiomes of CRS patients. Several bacteria in UT and NP tissues revealed an association with clinical severity of CRSwNP.

## Introduction

Chronic rhinosinusitis (CRS) is a common inflammatory upper airway disease that affects approximately 12.1% of the US population [1] and 8.4% of the Korean population [2]. CRS is clinically defined based on the presence of symptoms for over three months, which include positive endoscopic and/or computed tomography (CT) findings. CRS can be further classified according to the presence of nasal polyps (NPs) as CRS with NP (CRSwNP) and CRS without NP (CRSsNP) [3].

Clinically, it is important to differentiate between CRSwNP and CRSsNP, as patients with the former have a greater disease burden than those suffering from the latter in terms of disease severity and poor treatment outcomes [4, 5]. Additionally, CRSwNP and CRSsNP are known to have distinct biological characteristics, indicating the diverse pathogenesis of CRS as depicted by different inflammatory profiles [6].

Numerous studies have examined the role of commensal bacterial communities in CRS development. Interactions between the host immune system and sinonasal bacteria implicate microbiota in disease pathogenesis. For example, a well-known association exists between the *Staphylococcus aureus* superantigen IgE and Th2 inflammation in NPs [7, 8]. Additionally, *Pseudomonas aeruginosa* is known to be associated with a poorer quality of life and unfavorable outcomes in patients with CRS [9, 10]. However, the roles of other diverse bacteria in the pathogenesis of CRS remain unclear.

Although recent studies of the nasal microbiome have described the overall composition and dysbiosis of the microbiome in CRS [11–14], individual microbial data vary between studies. This discrepancy could be explained by diverse technical approaches and sampling methods utilized in each study, different anatomical sampling sites, and heterogenous patient distribution, such as different proportions of CRSwNP and CRSsNP.

As a proof of concept study, we hypothesized that microbiome in chronic rhinosinusitis could be affected by 1) the sampling method, 2) disease subtype, and 3) anatomical location of the sampling site. Therefore, in this study, nasal microbiota was analyzed in patients with CRS according to the sampling method (swab vs. tissue), disease subtype (with or without NPs), and tissue type [uncinate tissue (UT) vs. NP]. Considering the potential role of bacteria in the pathogenesis of CRS and the distinct characteristics of CRSwNP compared to CRSsNP, the microbiome profiles in the NP are likely to have distinct characteristics.

## Materials and methods

### Study participants

This cross-sectional study was based on the nasal microbiome profiles of patients who underwent endoscopic sinus surgery at Seoul National University Hospital from December 2015 to August 2016. Inclusion criteria was refractory primary bilateral CRSwNP and CRSsNP and the diagnosis was based on the European Position Paper on Rhinosinusitis with NPs [3]. Patients with unilateral disease, fungal disease, antrochoanal polyps, cystic fibrosis, primary ciliary dyskinesia, or other tumorous conditions, such as sinonasal inverted papilloma, were excluded. All patients were initially managed medically with three weeks of systemic antibiotics and intranasal corticosteroid treatment. Endoscopic sinus surgery was performed if no improvement was observed after initial therapy. Subjects who underwent endoscopic septoplasty for nasal-septal deviation without evidence of CRS were recruited as controls. Systemic steroids and antibiotics were avoided for at least four weeks before surgery. Twenty-two subjects were finally enrolled (8 CRSwNP patients, 6 CRSsNP patients, and 8 control subjects).

Demographic variables including age, sex, smoking history, atopy, asthma, antibiotic use, and intranasal steroid administration were evaluated for all patients. Patients were divided into current smoker and non-smoker. The non-smokers included patients who never smoked and those who had quit smoking at the time of interview. Patients who had been treated with systemic antibiotics within 6 months were counted. A complete blood cell count with differential was performed within four weeks prior to surgery to check the peripheral blood eosinophil count. Atopy was assessed by either a skin prick test to detect reactions to common inhalant allergens or a multiple allergen simultaneous test (Immunosystems, Mountain View, CA, USA). Patients presenting a positive response to methacholine inhalation or short-acting β2 agonists with a history of wheezing, shortness of breath, or chest tightness were regarded as having asthma [15]. Quantification of disease severity was analyzed by CT using the Lund-Mackay (LM) scoring system [16].

### Sample collection

Three different types of samples were acquired: 1) nasal swabs from the middle meatus, 2) tissue biopsied from the uncinate process (UT), and 3) NP tissue biopsied from the ethmoid sinus. All samples were collected intra-operatively. To eliminate the possibility of contamination, surgical draping with a solution composed of 2% chlorhexidine gluconate and 70% isopropyl alcohol was used for all patients. Swabs were taken from the middle meatus under endoscopic guidance with cotton ball swabs (Transystem™, COPAN, Brescia, Italy). After swabbing, the UT or NP was harvested surgically. All samples were collected by a single surgeon (D.Y. KIM). Samples were immediately separated into sterile containers, placed on ice, and transported to the laboratory for storage at -80°C. Any swabs that may have contacted the nasal vestibule were discarded. Finally, fifty-two samples from 22 patients in 3 different groups were collected (8 NPs, 22 UTs, and 22 swabs).

### DNA extraction, PCR amplification, and 16S rRNA gene sequencing

Swab heads were thawed, cut into small pieces, and then placed in 180 μL of enzymatic lysis buffer (Qiagen, Hilden, Germany) overnight at room temperature. Bead homogenization was performed with 5-mm steel beads agitated for 20 s at 15 Hz, followed by 0.1-mm glass beads for 5 min at 30 Hz. The same extraction procedure was carried out for the tissue biopsy samples. The remainder of the extraction protocol was performed using the FastDNA^®^ SPIN Kit for Soil DNA extraction according to the manufacturer’s instructions (MP Biomedicals, Irvine, CA, USA). Extracted DNA was stored at -80°C until sequencing. PCR amplification of the V3-V4 hypervariable region of the bacterial 16S rRNA gene was performed using 3 μL of the extracted DNA template and 25 μL 2X KAPA HiFi HotStart ReadyMix (KAPA Biosystems, Wilmington, MA, USA). The primers 318F (5′ TCGTCGGCAGCGTCAGATGTGTATAAGAGACAGCCTACGGGNGGCWGCAG-3′) and 806R (5′ GTCTCGTGGGCTCGGAGATGTGTATAAGAGACAGGACTACHVGGGTATCTAATCC-3′) were used for 16S rRNA gene amplification. The PCR cycling conditions were 3 min at 95°C; 25 cycles for 30 s at 95°C, 30 s at 55°C, and 30 s at 72°C; 5 min at 72°C; holding at 4°C. The amplicons were purified using Agencourt AMPure XP magnetic beads (Beckman Coulter, Brea, CA, USA). The purified products were subjected to index PCR using a Nextera Index Kit (Illumina, San Diego, CA, USA) following the manufacturer’s instructions. The products were further purified using AMPure XP beads. The resulting bacterial amplicon library was quantified, mixed with multiple libraries, and sequenced using the MiSeq v3 platform (Illumina).

### Sequence data processing

The raw sequence data files were preprocessed using several bioinformatics tools before downstream data analysis. To remove sequences with low quality scores, the raw reads were pre-filtered using PRINSEQ [17]. Adapter sequences were removed using CUTADAPT [18] and ambiguous bases were further trimmed using the FASTX-toolkit [19]. Pair-end reads were merged using PEAR [20] and further filtered with PRINSEQ. Chimeric sequences and singletons were screened and reduced using USEARCH [21]. A random sampling of 10000 reads was performed to equalize the sequencing depth between samples. Downstream data analysis was completed using QIIME [22] with the EzBiocloud 16S rRNA gene sequence database [23]. Operational taxonomic units were defined as clusters of sequences with ≥97% identity. Alpha-diversity indices were calculated using the core_diversity_analyses option. The weighted normalized UniFrac distance was calculated as beta-diversity and visualized using principal coordinate analysis (PCoA).

### Statistical analyses

Clinical parameters among each group were compared by Kruskal-Wallis tests with Dunn multiple comparisons tests and Fischer’s exact tests for categorical variables. Results are presented as median with interquartile range (IQR) for continuous variables. Alpha diversity metrics among each group were compared using the Kruskal-Wallis test and a *Wilcoxon signed*-*rank test*. The differential clustering of microbiomes depending on study groups were tested by permutational multivariate analysis of variance (PERMANOVA) using R program. To identify genera that best characterized UTs of each study group (control, CRSsNP, and CRSwNP), log-transformed bacterial relative abundance was regressed on the experimental groups after adjusting for the effect of age and sex using Rex version 3.0.3 (⁠RexSoft (2018). Rex: Excel-based statistical analysis software. URL http://rexsoft.org/.). To summarize distinct bacterial genera between the nasal polyp and uncinate tissue, linear discriminant analysis effect size (LEfSe) [24] was used. Taxa with linear discriminant analysis scores > 2 and *P* < 0.05 were considered to be significant. Simple linear regression was performed to identify the most relevant genera related to disease severity according to the LM score in CT scans. Spearman correlation coefficients were determined to assess the relationships between the relative abundance of genera and disease severity. Statistical analyses were performed using SPSS ver. 22.0 software (SPSS, Inc., Chicago, IL, USA). Simple linear regression was performed using Weka software [25]. Significance was accepted at *P* < 0.05 for multiple analyses.

### Ethics approval and consent to participate

All methods were carried out in accordance with the ethical standards of the 1964 Declaration of Helsinki and its later amendments. This study was approved by the institutional review board of Seoul National University Hospital (IRB-No. 1702-009-828). All participants and parents of minor participants gave written informed consent.

## Results

### Clinical characteristics of study participants

The age of the study participants was 21 to 76 years and male to female ratio was 12 to 10. Among the three groups, clinical parameters including age, male to female ratio, presence of atopy or asthma, usage of intranasal steroid or oral antibiotics, and smoking status did not differ, but pre-operative CT scores showed significant differences (*P* = 0.001, Table 1). Patients with CRSwNP (median 11.0, IQR 8.5) and patients with CRSsNP (median 10.5, IQR 12.0) had significantly higher CT scores than controls (median 0, IQR 0) (*P* = 0.02 and *P* = 0.027 for CRSwNP vs control and CRSsNP vs control, respectively). However, there was no significant difference between the average CT scores of CRSwNP and CRSsNP groups (*P* > 0.999).

**Table 1:** Clinical characteristics of study participants.

	CRSwNP	CRSsNP	Control	*P*-value
Number of samples	8	6	8	-
Age (years) (median, (IQR))	48 (30.8)	38 (27.3)	48 (30.8)	0.809
Male/female	3/5	5/1	4/4	0.222
Atopy	1/8	2/6	3/8	0.493
Asthma	2/8	0/6	0/8	0.146
Smoking	1/8	1/6	0/8	0.514
Nasal steroid	5/8	4/6	4/8	0.797
Preoperative antibiotics (within 6 months)	3/8	3/6	1/8	0.300
Lund-Mackay score (median, (IQR))	11.0 (8.5)	10.5 (12.0)	0 (0)	0.001
Blood eosinophil % (median, (IQR))	4.2 (6.4)	4.5 (2.3)	3.0 (3.8)	0.759

Abbreviations: CRSwNP, chronic rhinosinusitis with nasal polyp; CRSsNP, chronic rhinosinusitis without nasal polyp; IQR, interquartile range.

### Microbial composition varies based on sample sites and disease subtypes

**Fig 1:**
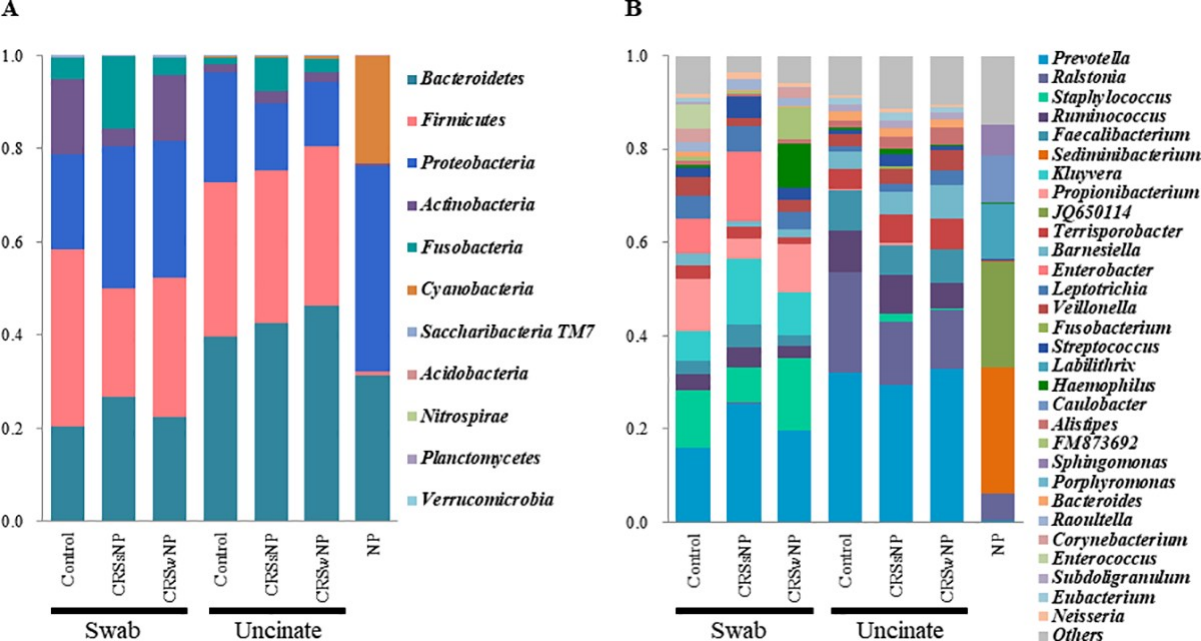
Distribution of bacterial taxa across disease and sampling subsites. Each stacked bar chart summarizes the percent relative abundances. A, At the phylum level, *Bacteroidetes*, *Firmicutes*, *Proteobacteria*, *Actinobacteria*, and *Fusobacteria* were the five dominant phyla in both swabs and uncinate tissues regardless of the disease subtype. B, Top 30 bacterial genera in the microbiomes according to sample and disease subtypes. Abbreviations: NP, nasal polyp; CRSwNP, chronic rhinosinusitis with nasal polyp; CRSsNP, chronic rhinosinusitis without nasal polyp.

The microbial composition from swabs and UT were compared among the three groups (control, CRSsNP, and CRSwNP; Fig 1). There was no significant difference in α-diversity (Shannon index) as a function of disease status for the swab and UT samples (S1 Fig).

At the phylum level, 11 bacterial phyla were detected in the nasal microbiome (Fig 1A). *Bacteroidetes*, *Firmicutes*, *Proteobacteria*, *Actinobacteria*, and *Fusobacteria* were the dominant phyla, comprising more than > 99% in both swabs and UT regardless of the disease status. The 30 most abundant genera in nasal microbiomes across sampling sites and disease subtypes are presented in Fig 1B. In general, *Prevotella* was the most common in both swabs and UT, but the other major abundant taxa differed between sample types; *Staphylococcus*, *Propionibacterium*, and *Kluyvera* were more abundant in swabs and *Ralstonia*, *Ruminococcus* and *Barnesiella* were more abundant in UT (S2 Fig).

### UT sampling provides homogenous microbial profiling associated with disease subtype

**Fig 2:**
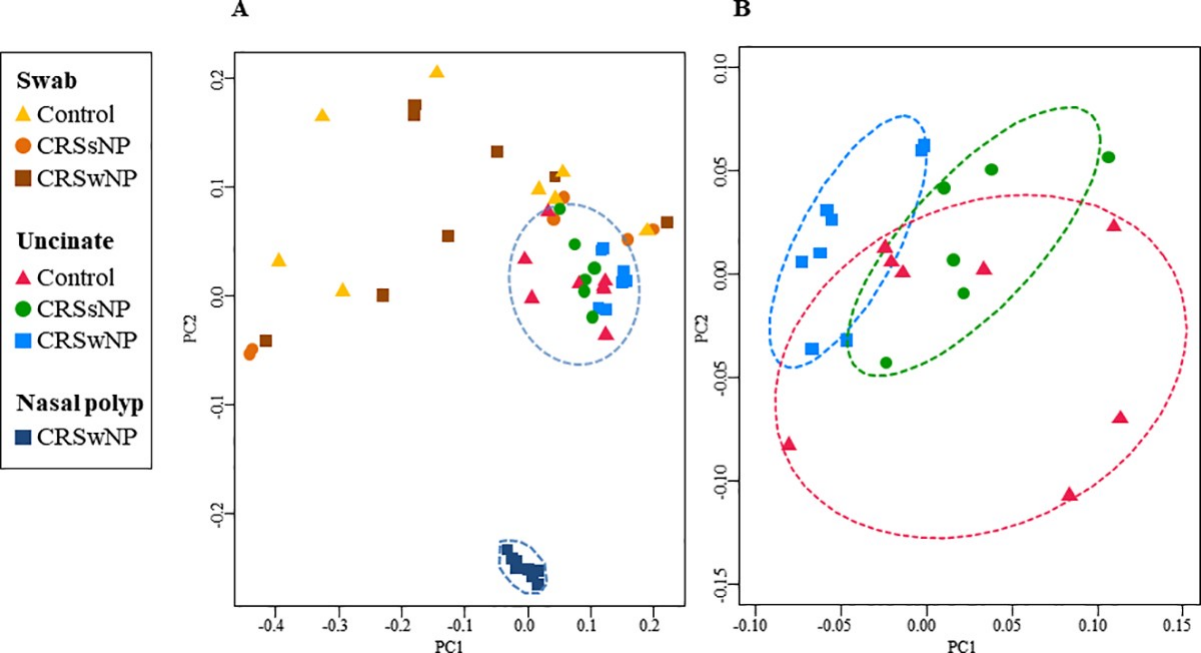
Variable microbiome structure as a function of clinical sample type and disease subtype. Beta-diversity was calculated by weighted UniFrac distance and plotted by PCoA. A, Microbiome distinctiveness depending on the clinical sample type. B, Uncinate tissue microbiome distinctiveness depending on the disease subtype. Confidence ellipse (dotted line) defines the region that contains 68% of all samples in each group. Abbreviations: CRSwNP, chronic rhinosinusitis with nasal polyp; CRSsNP, chronic rhinosinusitis without nasal polyp.

**Fig 3:**
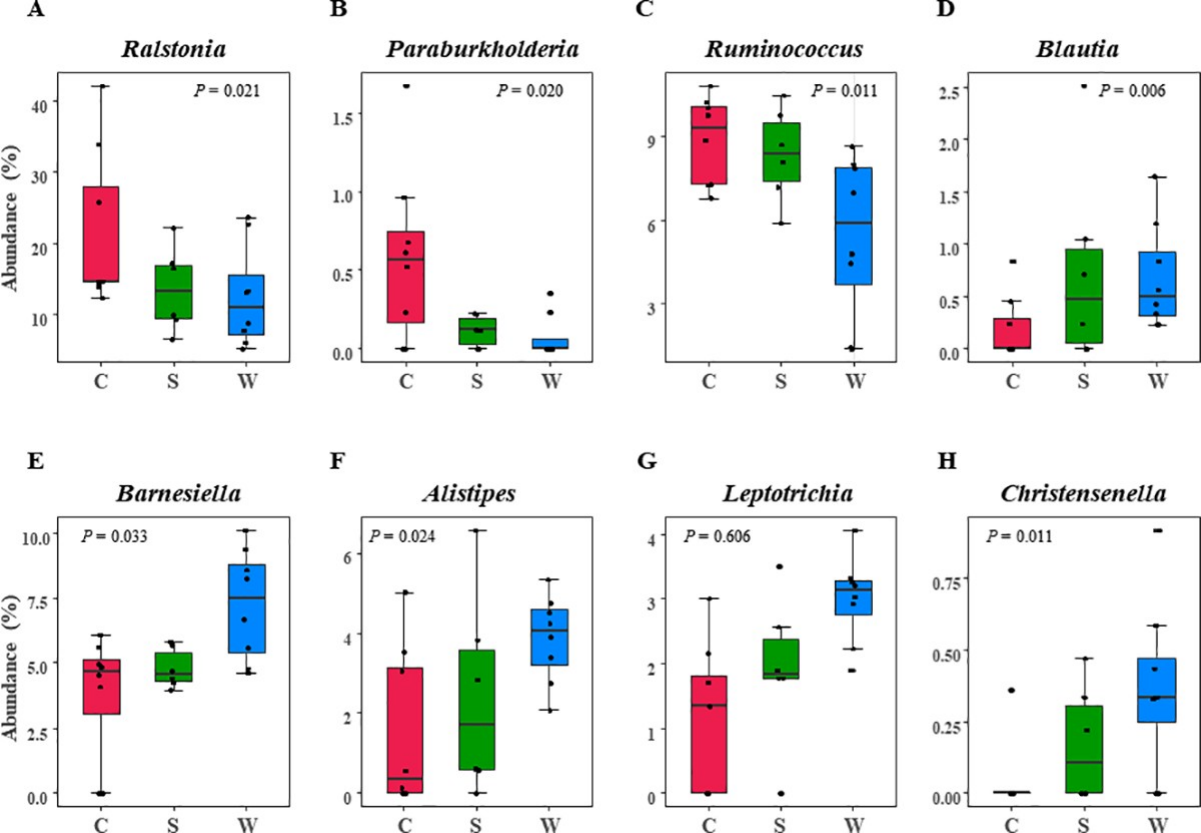
Uncinate tissue bacteria differ with disease subtype. Log-transformed bacterial genus abundances were regressed on the experimental groups to determine bacterial genera associated with disease subtypes. Only genera with *P* < 0.05 are shown. Abbreviations: C, control; S, CRSsNP (chronic rhinosinusitis without nasal polyp); W, CRSwNP (chronic rhinosinusitis with nasal polyp).

PCoA was performed based on the weighted UniFrac distances, which considers genus-level operational taxonomic unit data (Fig 2A). Nasal swab samples showed substantial in-group heterogeneity and microbial characteristics were not associated with specific disease subtypes. In contrast, UT formed a tight PCoA cluster, indicating the relative homogeneity of the microbiome in UT as compared to swabs. When the UT was further analyzed, a distinctive microbiome structure was observed depending on the disease subtype (Fig 2B). PERMANOVA using distance matrices revealed a significantly different microbial composition between the UT from patients with CRSwNP (UT_CRwNP) and those from both control subjects (UT_control, *P* = 0.006) and patients with CRSsNP (UT_CRSsNP, *P* = 0.003, Table 2). However, the microbiome of UT_CRSsNP did not significantly differ from that of UT_control according to PERMANOVA (*P* = 0.335). To identify bacterial genera associated with disease subtypes, log-transformed bacterial genus abundances was regressed on the experimental groups. The genera *Ralstonia*, *Ruminococcus* and *Paraburkholderia* showed decrement in the order of control, CRSsNP, to CRSwNP (Fig 3A–3C). On the other hand, the genera *Blautia*, *Barnesiella*, *Alistipes*, *Leptotrichia*, and *Christensenella* increased in the order of control, CRSsNP, to CRSwNP (Fig 3D–3H).

**Table 2:** The microbiome difference depending on disease subtype and tissue type.

**Disease subtype**	***P*-value**
UT_CRSwNP vs. UT_CRSsNP	0.003
UT_CRSwNP vs. UT_Control	0.006
UT_CRSsNP vs. UT_Control	0.335
**Tissue type**	***P*-value**
NP vs. UT_CRSwNP	0.002
NP vs. UT_Control	0.002

The P-values were calculated using permutational multivariate analysis of variance (PERMANOVA) using weighted UniFrac distance. Abbreviations: NP, nasal polyp; UT_CRSwNP, uncinate tissue from chronic rhinosinusitis with nasal polyp; UT_CRSsNP, uncinate tissue from chronic rhinosinusitis without nasal polyp.

### Characteristic dysbiosis in NP: Depletion of *Prevotella*

**Fig 4:**
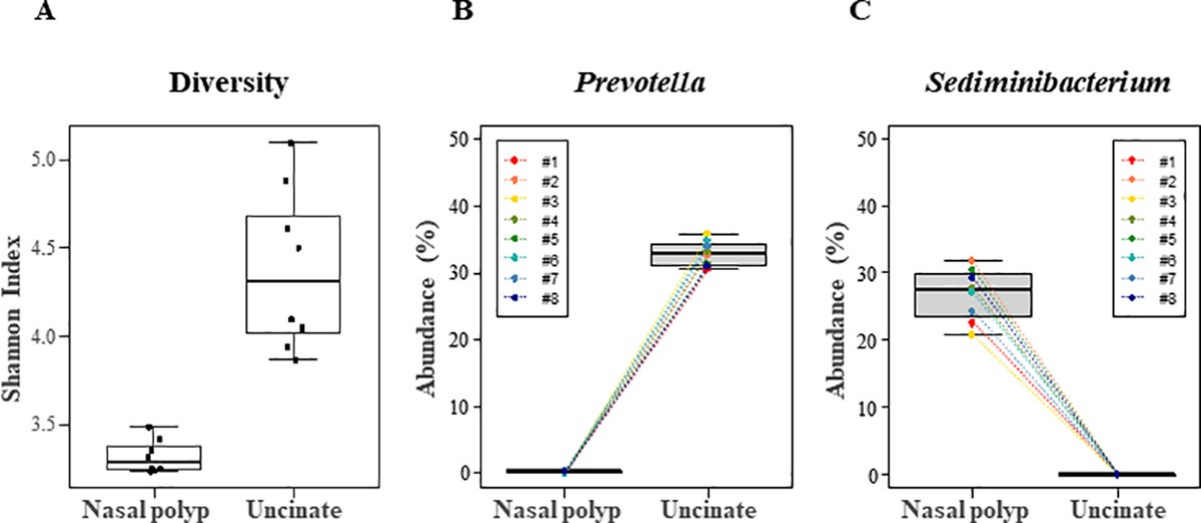
Characteristics of nasal polyp microbiome compared to uncinate tissue microbiome. Tissues from NP and UT of patients with CRSwNP were compared. A, NP exhibited a significantly decreased Shannon index (*P* = 0.012 by Wilcoxon signed rank test). B, The abundance of genus *Prevotella* is depleted in NP in all patients examined (n = 8). C, The abundance of genus *Sediminibacterium* is depleted in UT in all patients examined (n = 8). Abbreviations: NP, nasal polyp; UT, uncinate tissue; CRSwNP, chronic rhinosinusitis with nasal polyp.

The microbiomes of NP tissue from patients with CRSwNP were compared to those from the UT_CRSwNP and UT_control groups (S3 Fig). The Shannon index was significantly decreased in NP compared to UT_ CRSwNP, indicating decreased diversity in the NP (Fig 4A). PCoA further revealed a discrete microbiome structure in the NP as compared to the UT_ CRSwNP or UT_control microbiomes (Fig 2A). The PERMANOVA values demonstrated a significantly different microbial composition in the NP than in the UT_CRSwNP (*P* = 0.002) and UT_control (*P* = 0.002) groups (Table 2). The overall microbial taxa distribution of NP is depicted in Fig 1. The phylum *Firmicutes* was remarkably reduced in NP, whereas *Proteobacteria* were more abundant in NP than in UT. In contrast, *Cyanobacteria* accounted for less than 23.1% of bacteria in the NP but was less than 0.5% in the UT_CRSwNP and UT_control. At genus level, *Prevotella* was most predominant in the UT_CRSwNP (33.0%) and UT_control (32.1%) and was severely depleted to 0.3% in the NP (Fig 4B). In addition, *Ralstonia*, the second most common genus (16.1%), was reduced to 5.9% in the NP. Instead, *Sediminibacterium* (26.8%) the most predominant genus in the NP tissue was remarkably depleted to (0%) in UT tissue (Fig 4C). *Cyanobacterium_*JQ650114 (22.7%), *Labilithrix* (11.8%), *Caulobacter* (10.3%), and *Sphingomonas* (6.5%) were the next abundant genera in the NP tissue and all showed decrement in UT tissue. All bacterial genera whose abundances clearly differed between the NP and UT are summarized in S3 Fig.

### Microbiome composition is associated with disease severity

**Fig 5:**
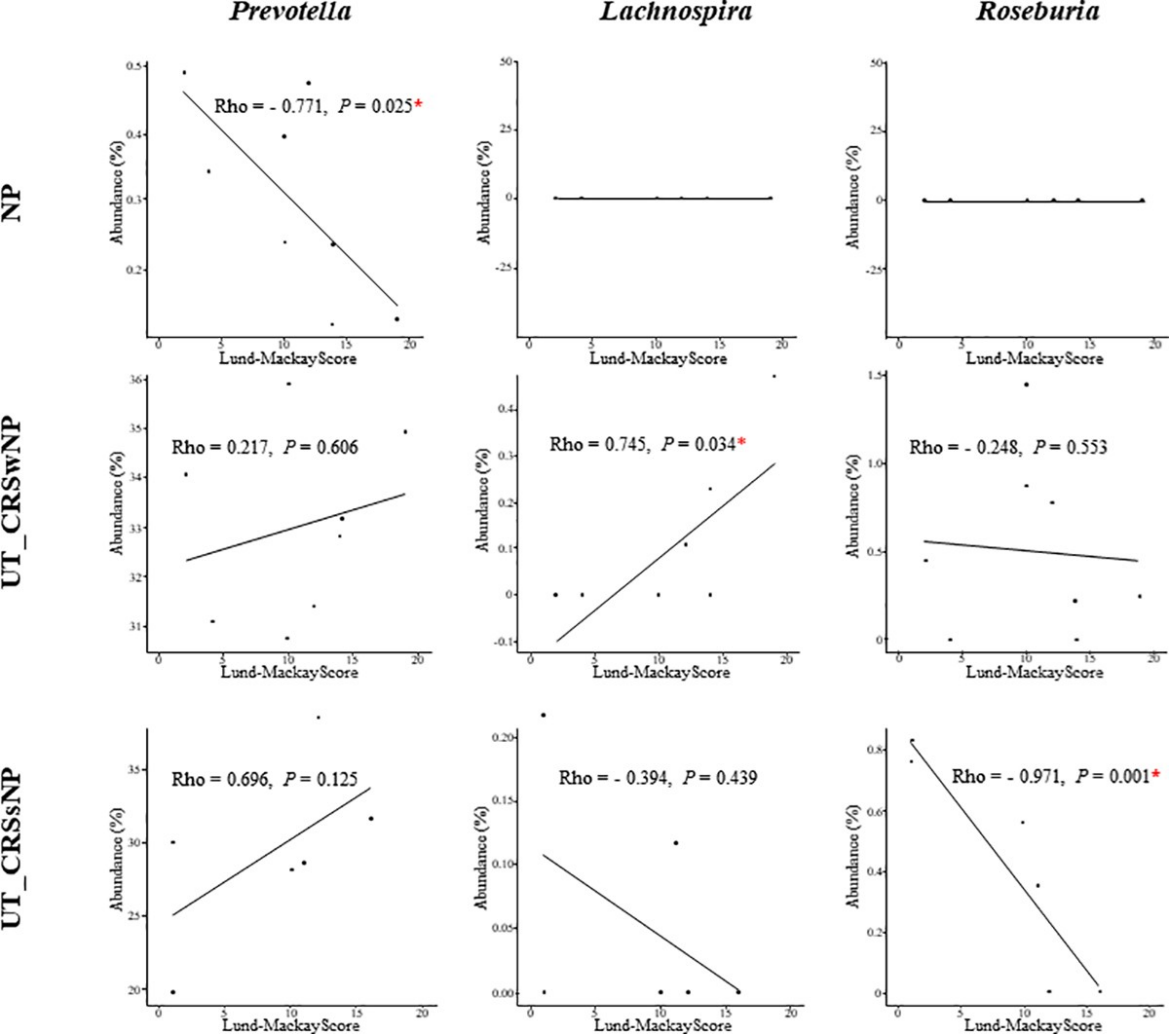
Correlation between bacterial genera and disease severity. The genera most correlated with disease severity in NP, UT_CRSwNP, and UT_CRSsNP were *Prevotella*, *Lachnospira*, and *Roseburia*, respectively. Relative abundances of these genera were then compared to Lund-Mackay scores by correlation analysis in different tissue microenvironments (NP, UT_CRSwNP, and UT_CRsNP). Abbreviations: NP, nasal polyp; UT, uncinate tissue; CRSwNP, chronic rhinosinusitis with nasal polyp; CRSsNP, chronic rhinosinusitis without nasal polyp; LM, Lund-Mackey.

A simple linear regression was performed to identify the genera most associated with disease severity using the LM score (Fig 5). The genera most associated with disease severity differed between tissue and disease subtypes. *Prevotella* was the genus most strongly inversely correlated to disease severity in NP tissue (Rho = -0.771, *P* = 0.025). However, *Lachnospira* (Rho = 0.745, *P* = 0.034) and *Roseburia* (Rho = -0.971, *P* = 0.001) were most associated with disease severity in the UT_CRSwNP and UT_CRSsNP, respectively. Several other genera in NP and UT associated with disease severity are summarized in S1 Table.

## Discussion

To date, most studies of CRS related microbiome have utilized nasal swabs, primarily collecting from the middle meatus. The middle meatus is an intersectional area that shares a common drainage pathway with other paranasal sinuses. Therefore, samples from this area may reflect multiple sites, which could produce increased heterogeneity. However, a study comparing microbial compositions from swab samples collected at different anatomical sites from patients with CRS still demonstrated a high interpersonal variation, which outweighed location-specific differences [14]. Our study found similar results, where a high interpersonal variability of swab samples may obscure the differences among disease subtypes.

In contrast, we demonstrated that tissue samples from the UT and NP may be more optimal specimens for assessing the microbiome of CRS than are nasal swabs. Tissues provided less variation between samples; moreover, in UTs different microbial compositions were associated with disease subtype. This allowed for the identification of discrete tissue-associated microbiomes. The high variation in swab samples could be a function of the vulnerability of the nasal surface microbiome to environmental changes such as temperature and humidity [26]. To our knowledge, this is the first study to use UT to assess the microbiome of patients with CRS. An advantage of using UT is the relative simplicity of sample collection without causing significant morbidity. Therefore, UT can be easily acquired regardless of disease status.

Our results are further supported by a previous study that found significantly different bacterial compositions in tissue specimens as compared to swab samples (30). Tissue specimens are more inclusive than swabs, as they incorporate bacterial biofilms that grow on the surface and bacteria that penetrate the mucosal epithelium. This is supported by the observation that tissue samples have a greater biomass than swabs, although non-microbial DNA is present (31). Thus, tissue samples should be given greater attention in the future as a collection method in studies of CRSwNP.

Several studies suggested an association with patient’s immunologic status and specific microbiome profile in CRS. For example, CRSwNP accompanied by asthma presented significantly higher levels of Th2-related cytokines and a higher abundance of *Proteobacteria* than in patients with CRSwNP without asthma [27]. However, we did not observe this relationship in this study due to the small sample size (only 2 showed asthma among the 8 CRSwNP patients). Copeland et al. demonstrated that patients with CRS can be categorized into 3 different subgroups defined based on the specific microbial composition with each exhibiting significantly different immune responses [28]. Therefore, different microbiome profile of UTs according to disease subtype or characteristic NP microbiome profile observed in the current study can be partially explained by their inflammatory profile. UTs in CRS studies has been demonstrated in its distinguishing immunological profile according to disease status. For instance, the eosinophil cationic protein (ECP) level in UT is strongly correlated with overall disease severity, comorbid asthma, and the risk of polyp recurrence [29, 30]. As for NP tissues, there is an upregulation of several important cytokines, such as IL-5, in NP compared to UT regardless of disease subtype [6, 31].

Our study identified several disease specific and tissue specific CRS microbiome. Among microbes in UT_CRSwNP, *Barnesiella* was one of the most distinguishing genus. *Barnesiella* belongs to Bacteroidetes and is known to be accumulated in the intestinal epithelium during chemotherapy in a NOD2 (Nucleotide-binding oligomerization domain 2) dependent manner. This accumulation further mediates immune reaction to cancer cell by reducing regulatory T cell [32]. Therefore, abundance of this implies a possibility of immunogenic reaction with the host in UT_CRSwNP. In the NP, the relative abundance of the phylum *Firmicutes* was remarkably lower than in the UT, whereas the abundance of the phylum *Proteobacteria* was higher. Higher abundance of *Proteobacteria* than that in controls has been consistently reported in other studies [28, 33, 34]. More specifically, which used different sampling method apart from tissue biopsy demonstrated that at the genus level, *Haemophilus*, *Escherichia*, and *Moraxella* were highly enhanced in CRSwNP as compared to controls. However, this had not been demonstrated in our study and instead, *Sediminibacterium*, a member of the phylum *Bacteroidetes*, was highly enriched in the NPs. Although there is a possibility of the reagent and laboratory contamination [35], depletion of this genus in the control tissue suggest the true residence in the nasal polyp tissue. Association of increased *Sediminibacterium* and disease had been demonstrated in patient with lung cancer [36], however interaction between host and this genus is unknown. Furthermore, in our study, *Sphingomonas*, a genus in the phylum *Proteobacteria*, was significantly enriched in the NP as compared to in the UT. In a murine asthma model, glycoproteins from *Sphingomonas* induced type 2 inflammation via natural killer T cells in an IL-4-, IL-13-, and IL-33-dependent manner [37, 38]. Interestingly, *Sphingomonas* was found to be more abundant in bronchoalveolar lavage fluid from patients with eosinophil-high asthma than in those with eosinophil-low asthma; this is also related to increased airway hyperresponsiveness to methacholine [39, 40]. In contrast, *Ralstonia* which was depleted in NP was enriched in UTs in the order of CRSwNP, CRSsNP, and control. This genus had been identified in human mesentery adipose tissue, and *Ralstonia* treated diet-induced obese mice had reduced glucose tolerance [41], which suggested potential pathogenic interactions between *Ralstonia* and host. In this study, *Ralstonia* depletion in NP tissues and its abundance in UTs, especially in normal control suggests a different role of *Ralstonia* in sinonasal tissues.

We also evaluated the severity of CRS using LM scores, a widely used radiological parameter assessed by CT. The score increases with certain markers of disease severity, such as the increasing grade of polyposis, nature of surgery offered (i.e. more extensive surgery), and treatment outcome [16]. Correlation between genera and disease severity differed by both tissue and disease subtype. This indicates unique interactions of microbiome according to the different microenvironmental conditions.

In the NP microbiome, the genus *Prevotella* was significantly and inversely correlated with disease severity. Similar findings have been observed in other inflammatory diseases including multiple sclerosis (MS). In that study, the abundance of gut *Prevotella* was reduced in untreated patients with MS, and treatment with disease-modifying therapy was associated with an increased relative abundance of *Prevotella* [42]. This protective effect of *Prevotella* appears to be mediated through the induction of CD4^+^ FoxP3^+^ regulatory T cells [43]. However, due to low abundance of this genus in NP, the role of *Prevotella* in pathogenesis of CRSwNP may be limited.

There were several limitations of our study. First, the number of patients was rather small, considering the heterogeneity of CRS. In addition, due to small sample size, factors which may affect the composition of sinus microbiome such as previous antibiotics usage and cigarette smoking were not considered. Second, we did not analyze the differences between eosinophilic NP and non-eosinophilic NP, which are known to exhibit immunological differences [31]. Third, 16S rRNA analysis only reveals the presence of bacteria, but it does not analyze their metabolic activity. Fourth, tissue samples dominated by non-microbial DNA can introduce contamination during amplicon sequencing. Therefore, further validation studies with larger sample sizes and adjustment of factors which may affect the microbiome composition are necessary. Future studies should also identify interactions between the tissue microenvironment and microbes in association with immunological profiles.

## Conclusions

Tissue microbiomes in CRS reveal highly clustered profiles with low inter-personal differences, with changes are associated with disease status. Most importantly, we identified a distinct NP microbiome composition as compared to UT. Therefore, tissue samples are encouraged to be used for future evaluation of CRS microbiomes. In all, the altered microbial habitat and strong association with CRS disease severity suggests a distinct tissue microenvironment that requires further study.

## Supporting information

S1 Fig Comparison of alpha diversity. Swab samples (SW) taken at middle meatus (A) and uncinate tissue samples (UT) (B) were compared according to disease subtype (control, CRSsNP, and CRSwNP). In both SW and UT, no significant differences were found in the Shannon index among disease subtype (*P* = 0.750 and *P* = 0.308 for SW and UT, respectively) based on Kruskal-Wallis test. Abbreviations–SW: Swab, UT: uncinate tissue, CRSsNP: chronic rhinosinusitis without nasal polyp, CRSwNP: chronic rhinosinusitis with nasal polyp.
(DOCX)

S2 Fig Distribution of bacterial taxa depending on sample types. Samples from different disease subtypes were averaged. A, *Bacteriodetes*, *Firmicutes*, *Proteobacteria*, *Actinobacteria*, and *Fusobacteria* were the five dominant phyla, comprising more than >99% in both swabs and UT regardless of the disease status. B, At the genus level, the overall profile of the genus composition differed between the swabs and uncinate tissues (UT). Although, *Prevotella* was the most common genus in both the swabs and UT, the second and third most abundant bacteria differed between samples types (*Staphylococcus* and *Propionibacterium* in swab and *Ralstonia* and *Ruminococcus* in UT).
(DOCX)

S3 Fig Linear discriminant analysis (LDA) demonstrates distinct bacterial genera between the nasal polyp and uncinate tissue. A, Nasal polyp vs. uncinate tissue from patients with chronic rhinosinusitis with nasal polyp (UT_CRSwNP) and B, nasal polyp vs. uncinate tissue from controls (UT_control).
(DOCX)

S1 Table Microbiome association with disease severity. Multiple genera associated with disease severity were identified using Lund-Makay CT score. Correlated genera were different between disease subtypes (CRSwNP or CRSsNP) and tissue types (nasal polyp or uncinate tissue). Abbreviations: CRSwNP, chronic rhinosinusitis with nasal polyp; CRSsNP, chronic rhinosinusitis without nasal polyp; RA, relative abundance.
(DOCX)

## References

[1] Blackwell DL, Lucas JW, TC C. Summary health statistics for U.S. adults: national health interview survey. Vital Health Stat 10. 2012;2014(260):1–161. 24819891

[2] Ahn JC, Kim JW, Lee CH, Rhee CS. Prevalence and Risk Factors of Chronic Rhinosinusitus, Allergic Rhinitis, and Nasal Septal Deviation: Results of the Korean National Health and Nutrition Survey 2008–2012. JAMA Otolaryngol Head Neck Surg. 2016;142(2):162–7. 10.1001/jamaoto.2015.3142 26747377

[3] Fokkens WJ, Lund VJ, Mullol J, Bachert C, Alobid I, Baroody F, et al. EPOS 2012: European position paper on rhinosinusitis and nasal polyps 2012. A summary for otorhinolaryngologists. Rhinology. 2012;50(1):1–12. 10.4193/Rhino50E2 22469599

[4] Banerji A, Piccirillo JF, Thawley SE, Levitt RG, Schechtman KB, Kramper MA, et al. Chronic rhinosinusitis patients with polyps or polypoid mucosa have a greater burden of illness. Am J Rhinol. 2007;21(1):19–26. 10.2500/ajr.2007.21.2979 17283555

[5] Toros SZ, Bölükbasi S, Naiboğlu B, Er B, Akkaynak C, Noshari H, et al. Comparative outcomes of endoscopic sinus surgery in patients with chronic sinusitis and nasal polyps. Eur Arch Otorhinolaryngol. 2007;246(9):1003–8. 10.1007/s00405-007-0301-5 17431658

[6] Stevens WW, Ocampo CJ, Berdnikovs S, Sakashita M, Mahdavinia M, Suh L, et al. Cytokines in Chronic Rhinosinusitis. Role in Eosinophilia and Aspirin-exacerbated Respiratory Disease. Am J Respir Crit Care Med. 2015;192(6):682–94. 10.1164/rccm.201412-2278OC 26067893 PMC4595675

[7] Tomassen P, Vandeplas G, Van Zele T, Cardell LO, Arebro J, Olze H, et al. Inflammatory endotypes of chronic rhinosinusitis based on cluster analysis of biomarkers. J Allergy Clin Immunol. 2016;137(5):1449–56. 10.1016/j.jaci.2015.12.1324 26949058

[8] Van Zele T, Gevaert P, Watelet JB, Claeys G, Holtappels G, Claeys C, et al. Staphylococcus aureus colonization and IgE antibody formation to enterotoxins is increased in nasal polyposis. =. J Allergy Clin Immunol. 2004;114(4):981–3. 10.1016/j.jaci.2004.07.013 15480349

[9] Bendouah Z, Barbeau J, Hamad WA, Desrosiers M. Biofilm formation by Staphylococcus aureus and Pseudomonas aeruginosa is associated with an unfavorable evolution after surgery for chronic sinusitis and nasal polyposis. Otolaryngol Head Neck Surg. 2006;134(6):991–6. 10.1016/j.otohns.2006.03.001 16730544

[10] Cleland EJ, Bassiouni A, Wormald PJ. The bacteriology of chronic rhinosinusitis and the pre-eminence of Staphylococcus aureus in revision patients. Int Forum Allergy Rhinol. 2013;3(8):642–6. 10.1002/alr.21159 23468020

[11] Biswas K, Hoggard M, Jain R, Taylor MW, Douglas RG. The nasal microbiota in health and disease: variation within and between subjects. Front Microbiol. 2015(9):134. 10.3389/fmicb.2015.00134 25784909 PMC4345913

[12] Choi EB, Hong SW, Kim DK, Jeon SG, Kim KR, Cho SH, et al. Decreased diversity of nasal microbiota and their secreted extracellular vesicles in patients with chronic rhinosinusitis based on a metagenomic analysis. Allergy. 2014;69(4):517–26. 10.1111/all.12374 24611950

[13] Lal D, Keim P, Delisle J, Barker B, Rank MA, Chia N, et al. Mapping and comparing bacterial microbiota in the sinonasal cavity of healthy, allergic rhinitis, and chronic rhinosinusitis subjects. Int Forum Allergy Rhinol. 2017;7(6):561–9. 10.1002/alr.21934 28481057

[14] Ramakrishnan VR, Gitomer S, Kofonow JM, Robertson CE, Frank DN. Investigation of sinonasal microbiome spatial organization in chronic rhinosinusitis. Int Forum Allergy Rhinol. 2017;7(1):16–23. 10.1002/alr.21854 27627048 PMC5218946

[15] Sistek D, Tschopp JM, Schindler C, Brutsche M, Ackermann-Liebrich U, Perruchoud AP, et al. Clinical diagnosis of current asthma: predictive value of respiratory symptoms in the SAPALDIA study. Swiss Study on Air Pollution and Lung Diseases in Adults. Eur Respir J. 2001;17(2):214–9. 10.1183/09031936.01.17202140 11334122

[16] Hopkins C, Browne JP, Slack R, Lund V, Brown P. The Lund-Mackay staging system for chronic rhinosinusitis: how is it used and what does it predict? Otolaryngol Head Neck Surg. 2007;137(4):555–61. 10.1016/j.otohns.2007.02.004 17903570

[17] Schmieder R, Edwards R. Quality control and preprocessing of metagenomic datasets. Bioinformatics. 2011;27(6):863–4. 10.1093/bioinformatics/btr026 21278185 PMC3051327

[18] Martin M. Cutadapt removes adapter sequences from high-throughput sequencing reads. EMBnet J. 2011;17(1):3.

[19] Gordon J, Hannon G. FASTX-Toolkit 2010 [Available from: http://hannonlab.cshl.edu/fastx_toolkit.

[20] Zhang J, Kobert K, Flouri T, Stamatakis A. PEAR: a fast and accurate Illumina Paired-End reAd mergeR. Bioinformatics. 2014;30(5):614–20. 10.1093/bioinformatics/btt593 24142950 PMC3933873

[21] Edgar RC. Search and clustering orders of magnitude faster than BLAST. Bioinformatics. 2010;26(19):2460–1. 10.1093/bioinformatics/btq461 20709691

[22] Caporaso JG, Kuczynski J, Stombaugh J, Bittinger K, Bushman FD, Costello EK, et al. QIIME allows analysis of high-throughput community sequencing data. Nat Methods. 2010;7(5):335–6. 10.1038/nmeth.f.303 20383131 PMC3156573

[23] Edgar RC. Search and clustering orders of magnitude faster than BLAST. Bioinformatics. 2010;26(19):2460–1. 10.1093/bioinformatics/btq461 20709691

[24] Segata N, Izard J, Waldron L, Gevers D, Miropolsky L, Garrett WS, et al. Metagenomic biomarker discovery and explanation. Genome Biol. 2011;12(6):R60. 10.1186/gb-2011-12-6-r60 21702898 PMC3218848

[25] Frank E, Hall MA, Witten IH, Workbench TW. Online appendix for “data mining: practical machine learning tools and techniques". 4 ed. Burlington: Morgan Kaufmann; 2016.

[26] Wagner Mackenzie B, Chang K, Zoing M, Jain R, Hoggard M, Biswas K, et al. Longitudinal study of the bacterial and fungal microbiota in the human sinuses reveals seasonal and annual changes in diversity. Sci Rep. 2019;9(1):17416. 10.1038/s41598-019-53975-9 31758066 PMC6874676

[27] Chalermwatanachai T, Vilchez-Vargas R, Holtappels G, Lacoere T, Jáuregui R, Kerckhof FM, et al. Chronic rhinosinusitis with nasal polyps is characterized by dysbacteriosis of the nasal microbiota. Sci Rep. 2018;8(1):7926. 10.1038/s41598-018-26327-2 29784985 PMC5962583

[28] Copeland E, Leonard K, Carney R, Kong J, Forer M, Naidoo Y, et al. Chronic Rhinosinusitis: Potential Role of Microbial Dysbiosis and Recommendations for Sampling Sites. Front Cell Infect Microbiol. 2018;8:57. 10.3389/fcimb.2018.00057 29541629 PMC5836553

[29] Min JY, Ocampo CJ, Stevens WW, Price CPE, Thompson CF, Homma T, et al. Proton pump inhibitors decrease eotaxin-3/CCL26 expression in patients with chronic rhinosinusitis with nasal polyps: Possible role of the nongastric H, K-ATPase. J Allergy Clin Immunol. 2017;139(1):130–41. 10.1016/j.jaci.2016.07.020 27717558 PMC5222859

[30] Weibman AR, Huang JH, Stevens WW, Suh LA, Price CPE, Lidder AK, et al. A prospective analysis evaluating tissue biopsy location and its clinical relevance in chronic rhinosinusitis with nasal polyps. Int Forum Allergy Rhinol. 2017;7(11):1058–64. 10.1002/alr.22005 28863237 PMC5966315

[31] Kim DK, Eun KM, Kim MK, Cho D, Han SA, Han SY, et al. Comparison Between Signature Cytokines of Nasal Tissues in Subtypes of Chronic Rhinosinusitis. Allergy Asthma Immunol Res. 2019;11(2):201–11. 10.4168/aair.2019.11.2.201 30661312 PMC6340796

[32] Daillère R, Vétizou M, Waldschmitt N, Yamazaki T, Isnard C, Poirier-Colame V, et al. Enterococcus hirae and Barnesiella intestinihominis Facilitate Cyclophosphamide-Induced Therapeutic Immunomodulatory Effects. Immunity. 2016;45(4):931–43. 10.1016/j.immuni.2016.09.009 27717798

[33] Jain R, Hoggard M, Zoing M, Jiang Y, Biswas K, Taylor MW, et al. The effect of medical treatments on the bacterial microbiome in patients with chronic rhinosinusitis: a pilot study. Int Forum Allergy Rhinol. 2018;8:890–9. 10.1002/alr.22110 29517178

[34] Taylor SL, Leong LEX, Choo JM, Wesselingh S, Yang IA, Upham JW, et al. Inflammatory phenotypes in patients with severe asthma are associated with distinct airway microbiology. J Allergy Clin Immunol. 2018;141(1):94–103.e15. 10.1016/j.jaci.2017.03.044 28479329

[35] Salter SJ, Cox MJ, Turek EM, Calus ST, Cookson WO, Moffatt MF, et al. Reagent and laboratory contamination can critically impact sequence-based microbiome analyses. BMC Biol. 2014;12:87. 10.1186/s12915-014-0087-z 25387460 PMC4228153

[36] Cheng C, Wang Z, Wang J, Ding C, Sun C, Liu P, et al. Characterization of the lung microbiome and exploration of potential bacterial biomarkers for lung cancer. Transl Lung Cancer Res. 2020;9(3):693–704. 10.21037/tlcr-19-590 32676331 PMC7354118

[37] Kim HY, Chang YJ, Subramanian S, Lee HH, Albacker LA, Matangkasombut P, et al. Innate lymphoid cells responding to IL-33 mediate airway hyperreactivity independently of adaptive immunity. J Allergy Clin Immunol. 2012;129(1):216–27. 10.1016/j.jaci.2011.10.036 22119406 PMC3246069

[38] Meyer EH, Goya S, Akbari O, Berry GJ, Savage PB, Kronenberg M, et al. Glycolipid activation of invariant T cell receptor+ NK T cells is sufficient to induce airway hyperreactivity independent of conventional CD4+ T cells. Proc Natl Acad Sci U S A. 2006;103(8):2782–7. 10.1073/pnas.0510282103 16478801 PMC1413796

[39] Huang YJ, Nelson CE, Brodie EL, Desantis TZ, Baek MS, Liu J, et al. Airway microbiota and bronchial hyperresponsiveness in patients with suboptimally controlled asthma. J Allergy Clin Immunol. 2011;127(2):372–81. 10.1016/j.jaci.2010.10.048 21194740 PMC3037020

[40] Sverrild A, Kiilerich P, Brejnrod A, Pedersen R, Porsbjerg C, Bergqvist A, et al. Eosinophilic airway inflammation in asthmatic patients is associated with an altered airway microbiome. J Allergy Clin Immunol. 2017;140(2). 10.1016/j.jaci.2016.10.046 28042058

[41] Udayappan SD, Kovatcheva-Datchary P, Bakker GJ, Havik SR, Herrema H, Cani PD, et al. Intestinal Ralstonia pickettii augments glucose intolerance in obesity. PLoS One. 2017;12(11):e0181693. 10.1371/journal.pone.0181693 29166392 PMC5699813

[42] Jangi S, Gandhi R, Cox LM, Li N, von Glehn F, Yan R, et al. Alterations of the human gut microbiome in multiple sclerosis. Nat Commun. 2016;7:12015. 10.1038/ncomms12015 27352007 PMC4931233

[43] Mangalam A, Shahi SK, Luckey D, Karau M, Marietta E, Luo N, et al. Human Gut-Derived Commensal Bacteria Suppress CNS Inflammatory and Demyelinating Disease. Cell Rep. 2017;20(6):1269–77. 10.1016/j.celrep.2017.07.031 28793252 PMC5763484

