# Supplementary material for: Microbiome profiling of uncinate tissue and nasal polyps in patients with chronic rhinosinusitis using swab and tissue biopsy
Source: PLoS One. 2021 Apr 8;16(4):e0249688. doi: 10.1371/journal.pone.0249688 (PMC8031401; doi:10.1371/journal.pone.0249688)
Supplement: S1 Fig — Swab samples (SW) taken at middle meatus (A) and uncinate tissue samples (UT) (B) were compared according to disease subtype (control, CRSsNP, and CRSwNP). In both SW and UT, no significant differences were found in the Shannon index among disease subtype (P = 0.750 and P = 0.308 for SW and UT, respectively) based on Kruskal-Wallis test. Abbreviations–SW: Swab, UT: uncinate tissue, CRSsNP: chronic rhinosinusitis without nasal polyp, CRSwNP: chronic rhinosinusitis with nasal polyp. (DOCX) [file pone.0249688.s001.docx]

**S1 Fig. Comparison of alpha diversity.** Swab samples (SW) taken at middle meatus (A) and uncinate tissue samples (UT) (B) were compared according to disease subtype (control, CRSsNP, and CRSwNP). In both SW and UT, no significant differences were found in the Shannon index among disease subtype (*P* = 0.750 and *P* = 0.308 for SW and UT, respectively) based on Kruskal-Wallis test. Abbreviations– SW: Swab, UT: uncinate tissue, NP: nasal polyp, CRSsNP: chronic rhinosinusitis without nasal polyp, CRSwNP: chronic rhinosinusitis with nasal polyp.

**
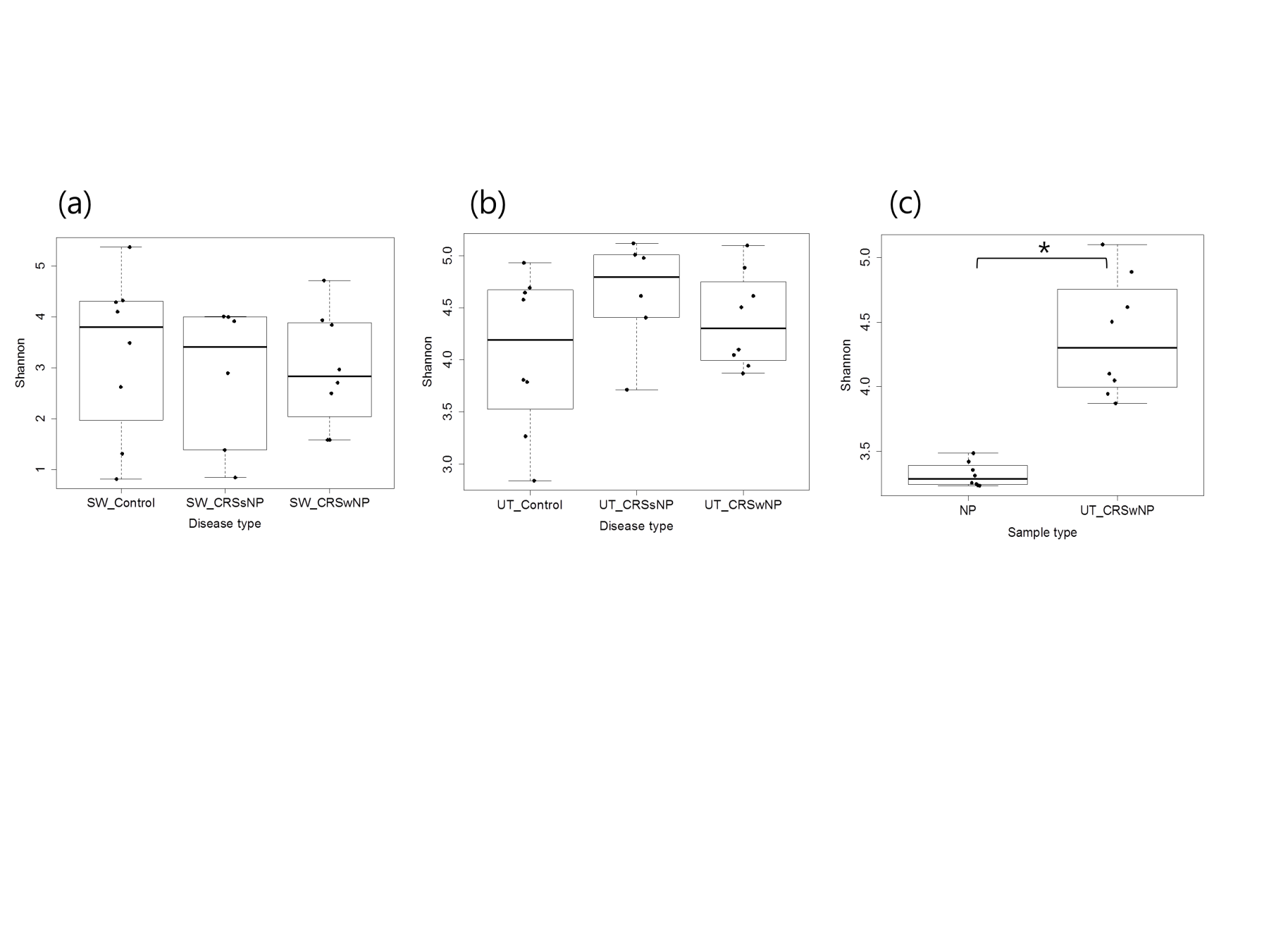
A B**
