# Supplementary material for: Microbiome profiling of uncinate tissue and nasal polyps in patients with chronic rhinosinusitis using swab and tissue biopsy
Source: PLoS One. 2021 Apr 8;16(4):e0249688. doi: 10.1371/journal.pone.0249688 (PMC8031401; doi:10.1371/journal.pone.0249688)
Supplement: S2 Fig — Samples from different disease subtypes were averaged. A, Bacteriodetes, Firmicutes, Proteobacteria, Actinobacteria, and Fusobacteria were the five dominant phyla, comprising more than >99% in both swabs and UT regardless of the disease status. B, At the genus level, the overall profile of the genus composition differed between the swabs and uncinate tissues (UT). Although, Prevotella was the most common genus in both the swabs and UT, the second and third most abundant bacteria differed between samples types (Staphylococcus and Propionibacterium in swab and Ralstonia and Ruminococcus in UT). (DOCX) [file pone.0249688.s002.docx]

**S2 Fig.** **Distribution of bacterial taxa depending on sample types**. Samples from different disease subtypes were averaged. A, *Bacteriodetes, Firmicutes, Proteobacteria, Actinobacteria,* and *Fusobacteria* were the five dominant phyla, comprising more than >99% in both swabs (SW) and uncinate tissues (UT) regardless of the disease status. B, At the genus level, the overall profile of the genus composition differed between the swabs and UT. Although, *Prevotella* was the most common genus in both the swabs and UT, the second and third most abundant bacteria differed between samples types (*Staphylococcus* and *Propionibacterium* in swab and *Ralstonia* and *Ruminococcus* in UT).

**
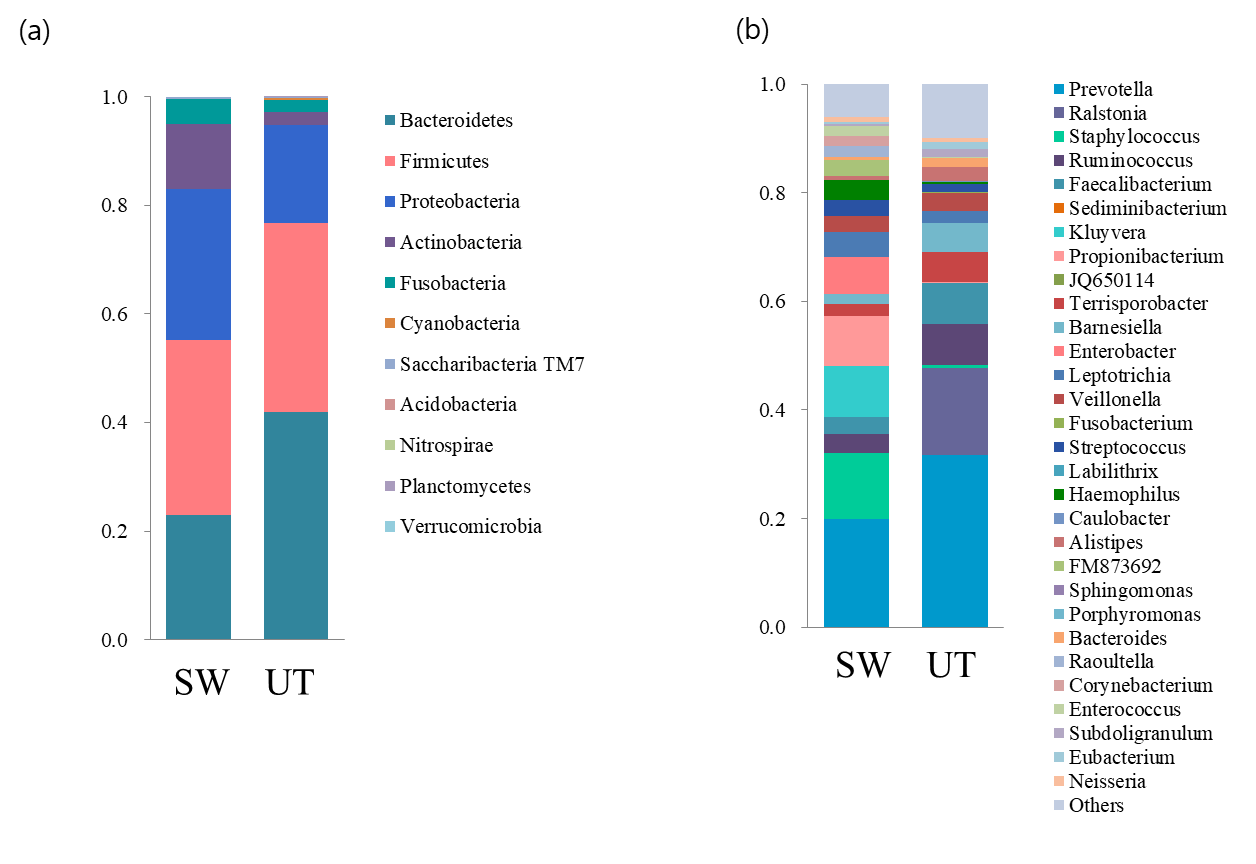
A B**
