# Supplementary material for: Microbiome profiling of uncinate tissue and nasal polyps in patients with chronic rhinosinusitis using swab and tissue biopsy
Source: PLoS One. 2021 Apr 8;16(4):e0249688. doi: 10.1371/journal.pone.0249688 (PMC8031401; doi:10.1371/journal.pone.0249688)
Supplement: S3 Fig — A, Nasal polyp vs. uncinate tissue from patients with chronic rhinosinusitis with nasal polyp (UT_CRSwNP) and B, nasal polyp vs. uncinate tissue from controls (UT_control). (DOCX) [file pone.0249688.s003.docx]

**S3 Fig. Linear discriminant analysis (LDA) demonstrates distinct bacterial genera between the nasal polyp and uncinate tissue.** A, Nasal polyp vs. uncinate tissue from patients with chronic rhinosinusitis with nasal polyp (UT_CRSwNP) and B, nasal polyp vs. uncinate tissue from controls (UT_control).

**A B
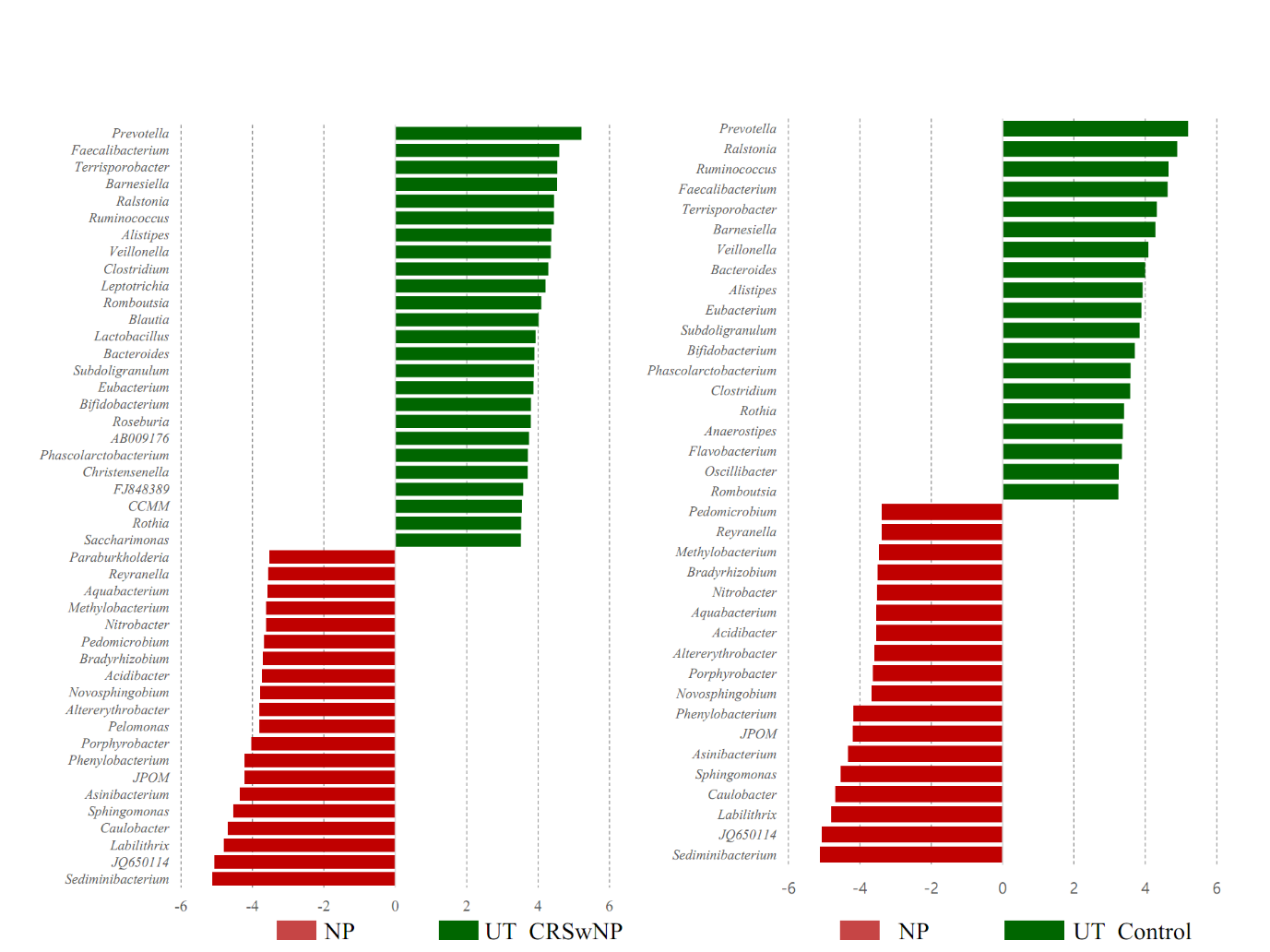
**
