# Supplementary material for: Microbiome profiling of uncinate tissue and nasal polyps in patients with chronic rhinosinusitis using swab and tissue biopsy
Source: PLoS One. 2021 Apr 8;16(4):e0249688. doi: 10.1371/journal.pone.0249688 (PMC8031401; doi:10.1371/journal.pone.0249688)
Supplement: S1 Table — Multiple genera associated with disease severity were identified using Lund-Makay CT score. Correlated genera were different between disease subtypes (CRSwNP or CRSsNP) and tissue types (nasal polyp or uncinate tissue). Abbreviations: CRSwNP, chronic rhinosinusitis with nasal polyp; CRSsNP, chronic rhinosinusitis without nasal polyp; RA, relative abundance. (DOCX) [file pone.0249688.s004.docx]

**S1 Table.** Microbiome association with disease severity. Multiple genera associated with disease severity by Lund-Makay CT score were identified. Correlated genera were different between disease subtypes (CRSwNP or CRSsNP) and tissue types (nasal polyp or uncinate tissue).

| **Disease subtype** | **Tissue type** | **Genus** | **Linear regression** | **Correlation coefficient** | **Spearman's rho** | **P-value** |
| --- | --- | --- | --- | --- | --- | --- |
| CRSwNP | Nasal polyp | *Prevotella* | -2732.02*RA+18.87 | 0.7119 | -0.771 | 0.025 |
|  |  | *Finegoldia* | 6484.72*RA + 8.64 | 0.6641 | 0.718 | 0.045 |
| CRSwNP | Uncinate tissue | *Lachnospira* | 2398.59*RA + 8.21 | 0.7366 | 0.745 | 0.034 |
|  |  | *Bacteroides* | -298.69*RA + 14.97 | 0.6972 | -0.807 | 0.015 |
|  |  | *Paraburkholderia* | 2780.38*RA + 8.61 | 0.6944 | 0.718 | 0.045 |
|  |  | *Agathobacter* | 3121.39*RA + 7.96 | 0.6653 | 0.745 | 0.034 |
| CRSsNP | Uncinate tissue | *Roseburia* | -1548.44*RA + 14.94 | 0.9142 | -0.971 | 0.001 |
|  |  | *Lachnoanaerobaculum* | -2944.35*RA + 11.8 | 0.8859 | -0.823 | 0.044 |
|  |  | AB185816 | -1177.74*RA + 11.8 | 0.8859 | -0.823 | 0.044 |
|  |  | *Rothia* | -2349.65*RA + 18.21 | 0.8817 | -0.928 | 0.008 |
|  |  | *Streptococcus* | -234.27*RA + 14.59 | 0.8713 | -0.899 | 0.015 |

Abbreviations: CRSwNP, chronic rhinosinusitis with nasal polyp; CRSsNP, chronic rhinosinusitis without nasal polyp; RA, relative abundance.
